# Prediction of Relapse of Acute Myeloid Leukemia based on Chest CT Features using a Nomogram

**DOI:** 10.2174/0115734056473256260826123151

**Published:** 2026-09-11

**Authors:** Shuying Li, Ting Li, Gang Wu

**Affiliations:** 1 School of Medical Imaging, Guizhou Medical University, Guiyang, Guizhou Province, China; 2 Department of Radiology, The Central Hospital of Wuhan, Tongji Medical College Affiliated to Huazhong University of Science and Technology, Wuhan, 430030, China; 3 Department of Radiology, Tongji Hospital, Tongji Medical College, Huazhong University of Science and Technology, Wuhan, China

**Keywords:** Nomogram, Acute myeloid leukemia, Chest CT, Radiomics, Automatic segmentation, Relapse-free survival, Minimum residual disease

## Abstract

**Introduction/Objective:**

To develop a novel model for predicting relapse risk in patients with acute myeloid leukemia (AML) based on chest CT features.

**Methods:**

A total of 131 patients from Wuhan Tongji Hospital and 30 patients from Wuhan Central Hospital with AML received standardized treatment and chest CT. The automatic segmentation of 51 organs was completed with TotalSegmentator. Follow-up on the survival and recurrence status of patients was performed to obtain the relapse-free survival (RFS) after CT examination. Feature selection was performed based on reproducibility, significant differences between patients with short and long RFS cases, and Least Absolute Shrinkage and Selection Operator (LASSO) regression. Cox proportional hazards regression was performed based on selected features. A nomogram was constructed and was to predict outcomes in the external validation cohort. Concordance index (C-index) and time-dependent area under the curve (AUC) were calculated for the model.

**Results:**

RFS and 5457 features were obtained for each case. After False discovery rate (FDR) control, the short and long RFS cases differed in 27 features. By using LASSO-Cox and initial Cox proportional hazards regression, 5 features were used in the final Cox model. In the external cohort, the C-index of the prediction model was 0.71. The time-dependent AUCs were 0.70, 0.71, 0.80, and 0.85, respectively, for the time points of 10, 20, 30, and 40 months.

**Discussion:**

Minimum residual disease (MRD) is closely related to relapse of AML. In the study, some CT features seemed to correlate with RFS of AML. A possible explanation is that these features reflect MRD in different organs. The nomogram displays the numerical correlations between variables through geometric shapes, and converts predictive indicators into visual scale segments based on regression models. The column chart used in this study helps to calculate the risk quickly.

**Conclusion:**

A total of 5 CT features from the heart, pulmonary venous system, T8, T11 vertebra, and adrenal gland were useful in predicting AML RFS. A nomogram was useful and convenient in calculating the risk.

## 1 INTRODUCTION

Acute myeloid leukemia (AML) is a malignant disorder of myeloid hematopoietic stem/progenitor cells [1, 2]. It is characterized primarily by the abnormal proliferation of immature myeloid cells in the bone marrow and peripheral blood, manifesting clinically as anemia, bleeding, infections, fever, and metabolic abnormalities [3, 4]. Most cases progress rapidly and have a poor prognosis. Induction remission therapy includes drugs such as daunorubicin, idarubicin, aclacinomycin, mitoxantrone, podophyllotoxins, doxorubicin, homoharringtonine, amsacrine, and thioguanine. Consolidation therapy is currently considered to involve early intensification with high-dose cytarabine to reduce late relapse [5]. When leukemia cells exceed 5% in the bone marrow, it is referred to as intramedullary relapse. Generally, about 50% of cases relapse within the first year after remission, while 80% relapse within three years.

Patients with AML sometimes undergo chest CT scans to rule out possible lung infections. Infiltration lesions of leukemia can also be revealed by high spatial resolution chest CT [6-8]. However, the value of chest CT in predicting relapse-free survival (RFS) of AML is not yet clear. Recently, the development of artificial intelligence has made automatic segmentation of multiple organs in chest CT fast and accurate. The TotalSegmentor with the core of nnUNet was preferred. On real-world chest CT, TotalSegmentor performed significantly better than single-organ models. TotalSegmentor combines strong algorithms, big data, clinical knowledge, and engineering optimization, and achieves high-precision, robust, and easy-to-use automatic segmentation on chest CT, suitable for scientific research and clinical translation.

After automatic segmentation is completed, radiomics features can be extracted for each organ [9, 10]. In this way, thousands of radiomics features can be obtained from chest CT images to reflect the microstructure and function of various organs. There is currently no literature reporting whether these features are correlated with RFS in AML patients. The purpose of the study is to determine which features of the chest CT image are useful in predicting RFS of AML and to establish a prediction model with the nomogram method.

## 2 MATERIALS AND METHODS

### 2.1 Patients

This is a retrospective study approved by the Institutional Review Board. The inclusion criteria were patients with AML and non-contrast-enhanced chest CT. The exclusion criteria were as follows: 1) unavailable RFS data; 2) absence of chest CT performed before relapse; 3) chronic obstructive pulmonary disease; 4) a history of chest surgery; 5) diabetes; 6) tuberculosis; 7) chronic cardiovascular disease; 8) rheumatic diseases; or 9) tumors other than AML. Altogether, 131 patients from Wuhan Tongji Hospital and 30 patients from Wuhan Central Hospital diagnosed with AML for the first time during August 2019 and September 2020 were included in the study. They received standardized treatment for acute myeloid leukemia in the hematology department.

### 2.2 Chest CT Examination

The main parameters of non-contrast chest computed tomography were as follows: anatomical coverage: extended to the upper abdomen to cover the adrenal gland; acquisition slice thicknesS: 0.625-1.25 mm; reconstruction interval: 50-80% of the acquisition slice thickness; tube voltage: 120 kVp; tube current (mA): automatic dose modulation; field of view: typically 35-40 cm; pitch: 1.0-1.5; matrix size: typically 512×512. Patients are instructed to inhale deeply and hold their breath during scanning to minimize respiratory motion artifacts.

The source CT data were transferred to a dedicated workstation with 3D slicer. The 3D slicer was equipped with the software of TotalSegmentator and Radiomics. The automatic segmentation of all organs was completed with TotalSegmentator. A total of 107 radiomics features were then extracted from each segmentation.

The following 51 organs were auto-segmented in the study: spleen, left kidney, liver, stomach, bilateral adrenal gland, esophagus, thyroid, twelve thoracic vertebrae, C7 vertebrae, heart, aorta, pulmonary venous system, bilateral humerus, bilateral scapula, bilateral clavicle, spinal cord, bilateral deep back muscle, 16 ribs (bilateral 3-10 ribs), sternum, and costal cartilage. As 107 radiomics features were extracted from each organ in the study, a total of 5457 features were obtained for each patient. The texture features included Gray Level Co-occurrence Matrix (GLCM), Gray Level Run-length Matrix (GLRLM), Gray Level Size Zone Matrix (GLSZM), Neighborhood Gray Level Difference Matrix (NGTDM), and Gray Level Dependence Matrix (GLDM). For the purpose of evaluating reproducibility, feature extraction was performed again one month later.

### 2.3 Relapse-free Survival

Follow-up on the survival and recurrence status of patients was performed through electronic medical records, outpatient follow-up, telephone follow-up, WeChat follow-up and other methods. The relapse of AML was defined according to the definition of the 2022 European Leukemia Working Group guidelines. If no relapse was observed, the follow-up should last 60 months. The RFS is defined as the interval between chest CT and the relapse or the interval between chest CT and the last follow-up.

### 2.4 Statistical Analysis

All statistical analysis was performed with Python. Intra-class correlation coefficients (ICC) were obtained for radiomics features. The features with ICC greater than 0.9 were considered stable and were compared between cases with short RFS and long RFS. A non-paired students’ t-test was used when the data obeyed normal distribution. If the data did not obey normal distribution, the Mann-Whitney U test was used. If the two groups differed significantly in a feature, it was considered significant. The false discovery rate (FDR) control method (Benjamini-Hochberg) was used for further selection of significant features. Then, the LASSO-Cox (Python CoxnetSurvivalAnalysis with L1_ratio=1.0) method was used for further feature selection. After LASSO-Cox, the coefficients of some features became zero. The initial Cox proportional hazards regression model was established based on the features with non-zero coefficients to identify independent risk factors.

By using the median of a feature as a threshold, cases were divided into two groups. The Kaplan-Meier test was performed for the two groups, and median RFS was compared between them by using the Log Rank test (python multivariate_logrank_test).

The final Cox model was established based on radiomics features, age, and gender. At last, the nomogram was constructed based on the coefficients of variables. The nomogram was then used for cases from Wuhan Central Hospital. The Concordance index (C-index) and time-dependent area under the curve (AUC) were calculated.

## 3 RESULTS

A total of 131 patients from Wuhan Tongji Hospital (median age, 52 years; male, 70) and 30 patients from Wuhan Central Hospital (median age, 53 years; male, 16) with AML underwent noncontrast-enhanced chest CT. Age, gender, RFS, and 5457 chest CT features were obtained for each case. The median of RFS was 24 months (95% confidence interval: 22, 26) for Wuhan Tongji Hospital cases. Kaplan-Meier and cumulative hazard curves were presented in Fig. (**1**). When cases were split with the median of Firstorder_Skewness_right_adrenal_gland, Glcm_Contrast_heart, Glszm_GrayLevelNonUniformityNormalized_pulmonary_venous_system, and Glszm_SmallAreaLowGrayLevelEmphasis_T8_vertebra, the two groups differed in median RFS (*p*<0.05, Table **1** and Fig. **2**).

The ICC for 4103 features was greater than 0.9. In the study, AML cases were divided into two groups: 1) with RFS shorter than 24 months and 2) equal to or longer than 24 months. The two groups differed in 125 out of 4103 features (*p*<0.05). After FDR correction, only 27 features were considered significant. By using LASSO-Cox, 13 features had non-zero coefficients, and they were used to establish the initial Cox proportional hazards regression model. In all, 5 features had *p*-values less than 0.05 in the initial model, so they were used for the final Cox model, as well as age and gender. Table **2** shows the results of Cox proportional hazards regression. Figure **3** is the nomogram using 7 variables to calculate the risk. The nomogram was then used for cases from Wuhan Central Hospital to generate predictions. By comparing the ground truth and predictions, the C-index was 0.71. The time-dependent AUCs were 0.70, 0.71, 0.80, and 0.85, respectively, for the time points of 10, 20, 30, and 40 months.

## 4 DISCUSSION

The study tried to identify independent risk factors of survival from chest CT features. The main findings were as follows: 1) several chest CT features were useful in predicting RFS in patients with AML; 2) a nomogram based on chest features could be used to estimate relapse risk.

In the present study, right adrenal gland skewness was selected as a predictor in the model for RFS in patients with AML. The adrenal gland has a very abundant blood supply and is easily infiltrated by leukemia cells. After treatment for acute myeloid leukemia, there may still be residual lesions in the adrenal gland, although they are not visible on CT or MR imaging. Skewness characterizes the degree of asymmetry in the data distribution [11, 12]. It is defined as a third-order standardized moment. The infiltration of leukemia cells may alter the skewness of the adrenal gland.

In the study, GLCM contrast from the heart seemed to correlate with RFS of AML. Leukemia is a malignant tumor of the hematopoietic system, mainly affecting the blood and bone marrow [13]. GLCM contrast reflects the total amount of local grayscale changes in the image. In an image, the greater the grayscale difference between local pixel pairs, the greater the image contrast and the clearer the visual effect of the image. As the concentration of leukemia cells changes, the appearance of blood undergoes minor changes.

In the study, Glszm_GrayLevelNonUniformityNormalized from the pulmonary venous system was selected in the model. The texture features represented by GLSZM mainly reflect the variability within the measurement area (heterogeneity of image texture), with lower values indicating stronger homogeneity between regions in the image. GLSZM is a counting matrix that stores the size and number of connected regions for all gray levels in the image. When GLSZM is wider and non-zero elements are concentrated on the right side, the texture of the original image is rougher. The more non-zero elements on the right side of GLSZM, the larger the area of the connected domain in the original image. GLSZM features of blood represent the coarseness of blood, which might be an indicator of leukemia cell concentration.

In the study, Glszm_SmallAreaLowGrayLevelEmphasis from the T8 vertebra seemed to correlate with RFS of AML. Some bones, such as thoracic vertebrae, retain red bone marrow for life. The presence of leukemia cells in bone marrow might alter the coarseness of thoracic vertebrae. Glszm_SmallAreaLowGrayLevelEmphasis may change with the concentration of leukemia cells in the vertebra.

It is well established that minimum residual disease (MRD) is closely related to relapse of AML [14]. In the study, some CT features seemed to correlate with RFS of AML. A possible explanation is that these features reflect MRD in different organs.

Nomograms are quantitative analysis charts that represent multivariate functional relationships in a plane coordinate system using families of non-intersecting line segments [15, 16]. It visually displays the numerical correlations between variables through geometric shapes and convert predictive indicators into visual scale segments based on regression models. The column chart used in this study helps quickly calculate the risk.

## 5 STUDY LIMITATIONS

The study has some limitations. First, the sample size is moderate. More cases should be collected in a future study. Second, MRD of AML was not measured by any methods. The correlation between the CT features and MRD is only at the level of speculation and has not been confirmed by invasive examinations. Third, clinical variables were not included in the prediction model. The focus was on the values of chest CT features.

## CONCLUSION

Altogether, 5 CT radiomics features from the heart, pulmonary venous system, T8, T11, vertebra, and adrenal gland were useful in predicting RFS in patients with AML. A model incorporating age, sex, and these 5 features could be established, and a nomogram provides a convenient means of estimating relapse risk.

## Figures and Tables

**Fig. (1):**
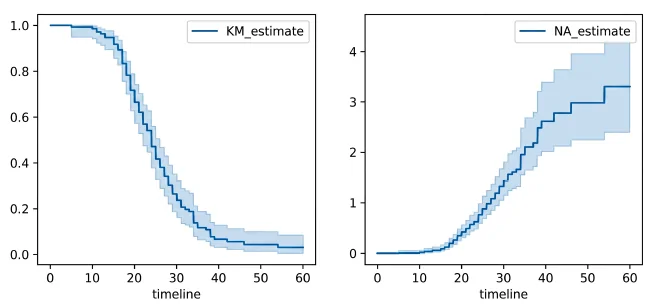
Kaplan-Meier and cumulative_hazard curves were presented for 131 cases from Wuhan Tongji Hospital.

**Fig. (2):**
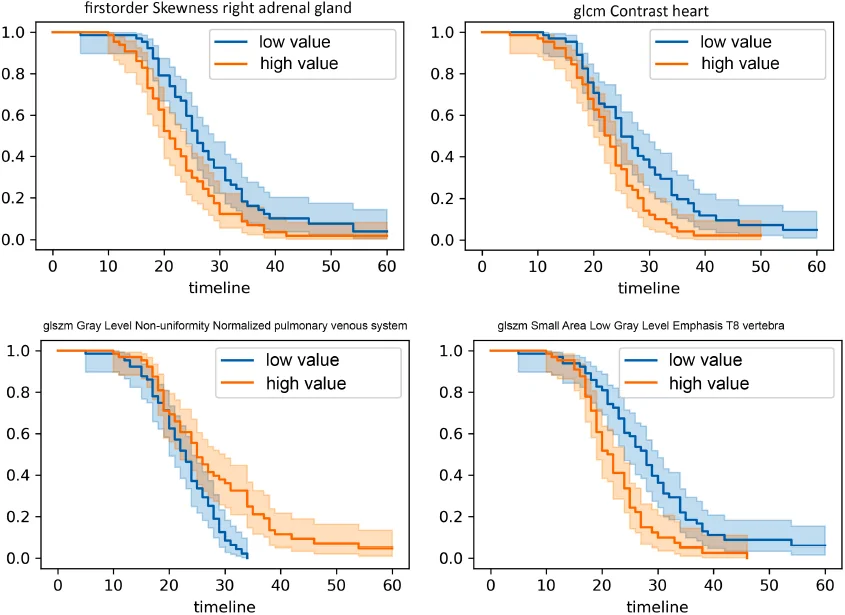
The comparison of survival curves between low value group and high value group. Firstorder_Skewness_right_adrenal_gland, Glcm_Contrast_heart, Glszm_GrayLevelNonUniformityNormalized_pulmonary_venous_system, and Glszm_SmallAreaLowGrayLevelEmphasis _T8_vertebra were respectively used as the classification variables.

**Fig. (3):**
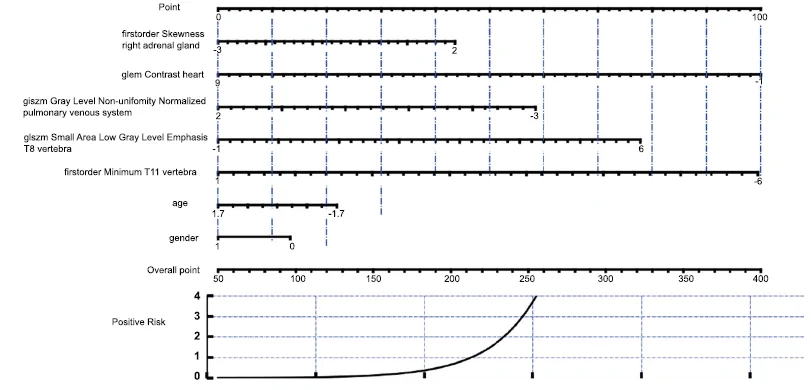
The risk can be calculated by using age, gender, Firstorder_Skewness_right_adrenal_gland, Glcm_Contrast_heart, Glszm_GrayLevelNonUniformityNormalized_pulmonary_venous_system, first_order_Minimum_T11_vertebra, and Glszm_SmallAreaLowGray LevelEmphasis_T8_vertebra.

**Table 1:** For 4 chest CT features, there is a significant difference in relapse-free survival between the low-value group and the high-value group.

-	**Min.**	**Median**	**Max.**	**Median of Low-value Group**	**Median of High- value Group**	***p*-value**
First-order skewness right adrenal gland	-1.174	-0.550	-0.050	26	21	0.015
GLCM contrast heart	0.602	1.528	12.338	25	23	0.016
glszm GrayLevelNonUniformityNormalized pulmonary venous system	0.035	0.057	0.086	23	25	0.002
glszm SmallAreaLowGrayLevelEmphasis T8 vertebra	0.001	0.002	0.008	28	21	0.000

**Table 2:** The results of the Cox proportional hazards regression model.

-	**Coef.**	**Exp(coef)**	**Lower**	**Upper**	***p*-value**
First-order skewness right adrenal gland	0.277	1.319	-3.184	2.545	0.005
GLCM contrast heart	-0.292	0.746	-1.025	9.064	0.015
glszm GrayLevelNonUniformityNormalized pulmonary venous system	-0.347	0.706	-2.255	2.900	0.006
glszm SmallAreaLowGrayLevelEmphasis T8 vertebra	0.347	1.415	-1.631	5.450	0.003
Firstorder minimum T11 vertebra	-0.442	0.642	-5.654	1.799	0.000
Age	-0.032	0.967	-1.648	1.707	0.734
Gender	-0.163	0.849	0	1	0.416

## Data Availability

Data regarding RFS and selected features are provided in supplementary material. The datasets generated and/or analyzed during the current study are available from the corresponding author, [G.W.], upon reasonable request.

## LIST OF ABBREVIATIONS

AML: Acute myeloid leukemia
RFS: Relapse-free survival
GLCM: Gray Level Co-occurrence Matrix
GLRLM: Gray Level Run-length Matrix
GLSZM: Gray Level Size Zone Matrix
NGTDM: Neighborhood Gray Level Difference Matrix
GLDM: Gray Level Dependence Matrix
ICC: Intra-class correlation coefficients
FDR: False discovery rate
C-index: Concordance index
AUC: Area under the curve
MRD: Minimum residual disease

## STANDARDS OF REPORTING

STROBE guidelines were followed.
